# Rewriting Viral Fate: Epigenetic and Transcriptional Dynamics in KSHV Infection

**DOI:** 10.3390/v16121870

**Published:** 2024-11-30

**Authors:** Chunyan Han, Danping Niu, Ke Lan

**Affiliations:** 1 State Key Laboratory of Virology, College of Life Sciences, Wuhan University, Wuhan 430072, China; chunyanhan@whu.edu.cn (C.H.); danpingniu@whu.edu.cn (D.N.); 2 Department of Infectious Diseases, Frontier Science Center for Immunology and Metabolism, Medical Research Institute, Zhongnan Hospital of Wuhan University, Wuhan University, Wuhan 430072, China; 3 Taikang Center for Life and Medical Sciences, Wuhan University, Wuhan 430072, China

**Keywords:** KSHV, life cycle, epigenetic modifications, transcriptional regulation

## Abstract

Kaposi’s sarcoma-associated herpesvirus (KSHV), a γ-herpesvirus, is predominantly associated with Kaposi’s sarcoma (KS) as well as two lymphoproliferative disorders: primary effusion lymphoma (PEL) and multicentric Castleman disease (MCD). Like other herpesviruses, KSHV employs two distinct life cycles: latency and lytic replication. To establish a lifelong persistent infection, KSHV has evolved various strategies to manipulate the epigenetic machinery of the host. In latently infected cells, most viral genes are epigenetically silenced by components of cellular chromatin, DNA methylation and histone post-translational modifications. However, some specific latent genes are preserved and actively expressed to maintain the virus’s latent state within the host cell. Latency is not a dead end, but the virus has the ability to reactivate. This reactivation is a complex process that involves the removal of repressive chromatin modifications and increased accessibility for both viral and cellular factors, allowing the activation of the full transcriptional program necessary for the subsequent lytic replication. This review will introduce the roles of epigenetic modifications in KSHV latent and lytic life cycles, including DNA methylation, histone methylation and acetylation modifications, chromatin remodeling, genome conformation, and non-coding RNA expression. Additionally, we will also review the transcriptional regulation of viral genes and host factors in KSHV infection. This review aims to enhance our understanding of the molecular mechanisms of epigenetic modifications and transcriptional regulation in the KSHV life cycle, providing insights for future research.

## 1. Introduction

Kaposi’s sarcoma-associated herpesvirus (KSHV), also known as human herpesvirus 8, was identified as a human cancer-associated virus in 1994 by Drs. Yuan Chang and Patrick Moore [1]. Various lymphocytic and epithelial malignancies can be directly associated with KSHV infection, including Kaposi’s sarcoma (KS), primary effusion lymphoma (PEL), multicentric Castleman disease (MCD), and an inflammatory syndrome known as KSHV inflammatory cytokine syndrome (KICS) [1,2,3,4]. KSHV is a large double-stranded DNA virus (~165 kb), and its genome encodes approximately 90 viral proteins and non-coding RNAs [5,6]. Like other herpesviruses, KSHV has a biphasic life cycle, a quiescent latent phase, and a lytic replication phase. During latency, the KSHV genome is transported into the cell nucleus, where it circularizes into a mini chromosome, known as an episome [7,8]. The episomal KSHV DNA resides in the nucleus bound to histone and non-histone proteins. This association indicates that epigenetic modifications—including DNA methylation, histone modifications, interactions with non-coding RNAs, and chromatin remodeling factors—may impact its activity and regulate viral gene expression. It has been suggested that only limited viral proteins and microRNAs are expressed during latency, including ORF73/LANA (latency-associated nuclear antigen), ORF72/v-Cyclin (virus-encoded homolog of human cyclin D), ORF71/v-FLIP (Fas-associated death domain-like interleukin-1β-converting enzyme-inhibitory protein) and K12/Kaposin family of protein (Kaposin A, B, and C) along with 25 mature microRNAs [9]. Latently infected cells can endure reactivation in response to various exogenous stimuli, which transitions the virus from latency to lytic replication [10,11]. Studies have indicated that inhibitors of histone deacetylases (HDACs) and DNA methyltransferases (DNMTs) can activate the expression of KSHV genes, highlighting the crucial roles of DNA methylation and histone modifications in gene regulation [11,12]. During lytic replication, the entire viral genome is expressed in a temporally regulated transcriptional cascade. This process features the sequential activation of three classes of lytic genes: immediate early (IE), early (E), and late (L) genes [13].

Besides epigenetic modifications that can alter the fate of KSHV, various viral and host transcriptional factors also play crucial roles in regulating the life cycle of KSHV. On one hand, differences in gene expression between the latent and lytic phases of KSHV are partially regulated by host transcription factors such as transcriptional co-activators and repressors. These factors, such as the nuclear factor kappa B (NF-κB) signaling pathway [14], specificity protein 1 (SP1) [15], activator protein 1 (AP-1) [16], signal transducer and activator of transcription 3 (STAT3) [17], octamer-binding transcription factor 1 (Oct-1) [18], Yin Yang 1 (YY1) [19], Krüppel-associated box domain-associated protein 1 (KAP1) [20], TLE2 [21], K-RBP [22], RUNX3 [23], etc., either facilitate or repress viral gene transcription. On the other hand, KSHV also deploys its own regulatory proteins, like LANA, vFLIP, RTA, and K-bZIP, and viral microRNAs, which are indispensable for either triggering lytic replication or regulating the viral latent–lytic cycle transition [10,16,24,25,26,27,28,29]. These interactions highlight a complex network of regulatory mechanisms that determine KSHV’s ability to persist and replicate within host cells, ultimately impacting pathogenesis and disease progression associated with this oncogenic virus. Understanding these factors is crucial for developing targeted therapies against KSHV-related diseases. This review will introduce the epigenetic regulation involved in KSHV infection, including DNA methylation, histone modifications, post-translational modifications, chromatin remodeling, genome conformation, and non-coding RNAs. In addition, this review will discuss the roles of both viral and host factors in regulating KSHV transcription.

## 2. Epigenetic Regulation in KSHV Infection

**Figure 1:**
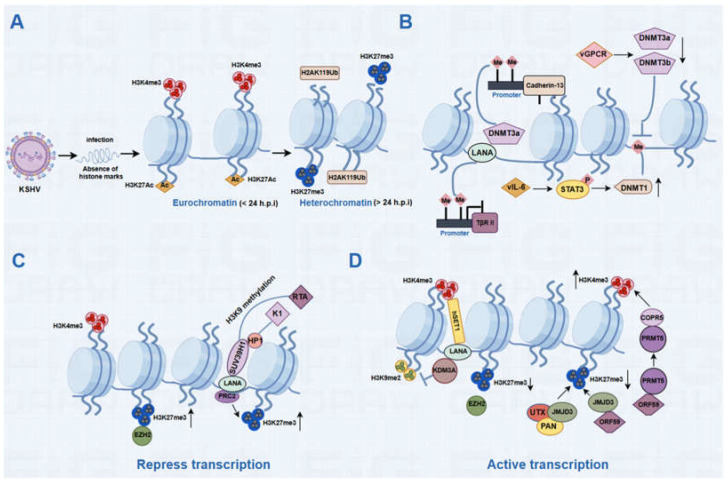
The chromatin landscape of the KSHV genome and the epigenetic regulatory mechanisms by which KSHV modulates its activity. (**A**) The chromatin landscape of the KSHV genome. During KSHV de novo infection, the initial viral genome is absent of histone marks. Within 24 h post-infection (hpi), the viral genome exhibits elevated levels of the activating histone marks H3K4me3 and H3K27ac. However, after 24 hpi, these activating marks begin to diminish, while the levels of repressive histone marks H3K27me3 and H2AK119ub increase significantly on the KSHV genome. (**B**) DNA methylation and KSHV infection: LANA interacts with DNMT3A, promoting the methylation and subsequent downregulation of the cadherin-13 promoter. LANA also binds to the promoter of the TGF-β type II receptor, inhibiting its expression through the induction of DNA methylation. vIL-6 enhances DNMT1 expression via STAT3 activation. vGPCR interacts with HIF1α and downregulates the expression of DNMT3A and DNMT3B. (**C**) Histone modifications repress KSHV transcription. Zeste homolog 2 (EZH2) binds to the KSHV genome and colocalizes with the repressive H3K27me3 mark to maintain KSHV latent infection. LANA recruits PRC2 to the KSHV genome and upregulates the level of H3K27me3 mark. In addition, LANA recruits and interacts with histone methyltransferase SUV39H1 and HP1 to induce H3K9 methylation of K1 and RTA, thus promoting the establishment of latent infection. (**D**) Histone modifications activate KSHV transcription. EZH2 dissociates from the KSHV genome, leading to downregulation of H3K27me3 mark to promote KSHV lytic replication. LANA interacts with the H3K9me1/2 demethylase KDM3A and promotes the removal of H3K9 methylation from the KSHV genome. LANA also interacts with the H3K4me3 methyltransferase hSET1, resulting in the deposition of H3K4me3 on the KSHV genome. PAN RNA interacts with H3K27me3 demethylases UTX and JMJD3 and removes H3K27me3 from the KSHV genome. ORF59 interacts with JMJD3, resulting in the decrease in H3K27me3. ORF59 also interacts with PRMT5 and disrupts its association with COPR5, leading to an increase in H3K4me3.

Epigenetic modifications can alter gene expression but do not change the underlying DNA sequence. These epigenetic modifications include DNA methylation, histone modifications, post-translational modifications, chromatin remodeling, genome conformation, and non-coding RNA expression. During viral infection, the viral genome undergoes epigenetic modifications that shape the transcriptional regulation of viral genes during both latency and lytic replication. These modifications also play a crucial role in the progression of their associated tumorigenesis. The chromatin landscape of the KSHV genome and the regulatory mechanisms by which viral proteins and host factors influence its activity through DNA methylation and histone modifications is summarized in Figure 1.

### 2.1. DNA Methylation and KSHV Infection

DNA methylation is a key player in the epigenetic regulation of eukaryotes. It tends to methylate cytosines throughout the majority of genomic DNA, with unmethylated cytosines predominantly found in specific regions known as CpG islands [30]. While most of the genome is CpG poor, some promoters contain high CpG content and are termed CpG islands. CpG islands control the expression of many housekeeping genes and are kept unmethylated in normal cells. However, in cancer cells, CpG islands often undergo methylation, altering their regulation patterns. Depending on the underlying gene sequence, DNA methylation in different genomic regions may exert different influences on gene activities. Generally, promoter methylation inhibits gene transcription, while gene body methylation induces gene transactivation [31]. DNA methylation is added and maintained by several DNA methyltransferases (DNMTs): DNMT1, DNMT3A, and DNMT3B. Several DNMTs are known to modulate the methylation of the KSHV genome. DNMT3A can interact with LANA and is recruited to chromatin, facilitating cadherin-13 promoter methylation and its downregulation, thus promoting the progression of Kaposi’s sarcoma [32] (Figure 1B). LANA also binds to the promoter of the transforming growth factor β (TGF-β) type II receptor and inhibit its expression by inducing DNA methylation [33] (Figure 1B). DNMT1 is upregulated by KSHV-encoded vIL-6 and increases the global genomic DNA methylation, thereby promoting cell proliferation and migration [34] (Figure 1B). Under hypoxic conditions, KSHV-encoded vGPCR interacts with hypoxia-inducible factor 1 alpha (HIF1α) and induces the production of reactive oxygen species, which in turn modulates the expression of DNMT3A and DNMT3B [35] (Figure 1B). In addition to viral proteins, KSHV-encoded microRNAs also regulate the expression of DNMTs. miR-K12-4-5p targets Rb12, which in turn increases the levels of DNMT3a, DNMT1, and DNMT3b and leads to CpG methylation of the KSHV episome [36] (Figure 1B).

Hijacking DNA methylation by KSHV is likely a mechanism by which the virus maintains latent infection. It has been shown that 5-azacytidine, a DNA methyltransferase inhibitor, can activate KSHV to enter lytic replication [12]. This finding highlights the importance of gene transcription de-repression as a key factor in initiating lytic replication. During latency, the promoter region of KSHV-RTA experiences extensive methylation. Demethylation of this region can boost RTA expression [12]. Additionally, an analysis of genome-wide methylation patterns in KSHV revealed widespread DNA methylation across the latent KSHV genome, with the exception of the latency-associated sites [37,38]. Subsequent CpG methylation analysis of KS tissues revealed widespread global methylation alterations in KS. Hypermethylation occurs in the early stages of KS, while hypomethylation occurs in the later stages. The dramatic changes between hypermethylation and hypomethylation on gene promoters and enhancers play a significant role in the regulation of abnormal skin morphology and tumorigenesis [39,40].

### 2.2. Histone Modifications and KSHV Infection

It has been shown that DNA methylation occurs later than histone modifications during KSHV latency [37]. Histones are core components of chromatin and undergo extensive modifications by diverse histone-targeting enzymes. These post-translational modifications exert significant influence on the accessibility of transcription factors and thus play a crucial role in regulating chromatin structure and gene expression. Histone modifications include methylation, acetylation, phosphorylation, ubiquitination, citrullination, etc. [41].

#### 2.2.1. Histone Methylation in KSHV Infection

In virions, the KSHV genome is histone-free [42,43]. However, once latency is established or following de novo infection, the viral genome rapidly acquires histone associations. A comprehensive genome-wide ChIP-on-Chip analysis of chromatin associated with the KSHV genome has shown that both activating and repressive histone marks colocalize on the latent genome [44]. During latency, the genomic regions encoding RTA and ORF48 present a distinctive chromatin landscape with the H3K4me3 mark, reflecting transcription activity and the H3K27me3 mark associated with transcription repression. This form of chromatin that harbors both activating and repressive histone marks is known as bivalent chromatin [45], and it provides the virus the ability to rapidly shift from repressed to activated promoter. Upon reactivation, there is a rapid increase in AcH3 and H3K4me3 marks and a decrease in the H3K27me3 mark. Notably, the presence of EZH2, a core component of the polycomb group protein complexes (PcG), has been implicated in the epigenetic regulation of KSHV. During latency, EZH2 binds to the KSHV genome, and colocalizes with the H3K27me3 mark. Upon reactivation, EZH2 dissociates from the viral genome, leading to a reduction in H3K27me3 levels, consequently triggering the initiation of lytic replication [44]. During de novo infection, biphasic chromatinization has been observed on the KSHV genome. Initially, the KSHV genome exhibited high levels of H3K4me3 and H3K27ac, accompanied by a transient activation of a limited number of lytic genes [46]. This phase was followed by a reduction in these activating marks and a shift toward an enrichment of repressive marks, particularly H3K27me3 and H2AK119ub [46]. This transition from euchromatin to heterochromatin has been found to depend on both polycomb repressive complex 1 and 2 (PRC1 and PRC2) [46].

KSHV-encoded LANA protein is essential for recruiting PRC1 and PRC2 to the KSHV genome following de novo infection, thus suppressing lytic gene expression [47]. In addition to its interactions with PRCs, LANA can also recruit specific methyltransferases and demethylases to the KSHV genome. Studies suggest that LANA interacts with and recruits the H3K4me3 methyltransferase hSET1 and the H3K9me1/2 demethylase KDM3A, contributing to the regulation of chromatin modifications on the KSHV genome [48,49]. This recruitment results in the deposition of H3K4me3 on the KSHV genome and the removal of H3K9 methylation from the KSHV genome, allowing LANA to bind more tightly on the KSHV genome [49]. LANA also recruits and interacts with histone methyltransferase SUV39H1 and heterochromatin protein 1, which facilitate the methylation of H3K9 on K1 and RTA [50]. This process promotes the establishment of latent infection. In addition to LANA, cellular factors may also be helpful to the recruitment of PRCs to the KSHV genome. For instance, the PRC component KDM2B directly binds to CpG islands, further facilitating the maintenance of KSHV latency [51,52].

Besides the methylation of lysine residues on histone H3, arginine methylation of histone H4 also plays a crucial role in regulating chromatin compactness. It has been suggested that the KSHV-encoded ORF59 interacts with protein arginine methyltransferase 5 (PRMT5), disrupting its association with COPR5 (cooperator of PRMT5). PRMT5 is responsible for the symmetric dimethylation of arginine 3 (R3) on histone H4. This disruption reduces the methylation of H4R3 mediated by PRMT5 and COPR5 while increasing the trimethylation of H3K4 (H3K4me3), thereby promoting the establishment of an open chromatin structure [53]. Additionally, ORF59 also interacts with KSHV long non-coding polyadenylated nuclear RNA (PAN RNA), which in turn associates with the cellular H3K27me3 demethylases UTX and JMJD3, effectively removing the repressive marks on the chromatin [54].

#### 2.2.2. Histone Acetylation in KSHV Infection

Acetylation and deacetylation play vital roles in regulating gene expression in eukaryotic cells. Histone acetylation is commonly associated with the activation of gene expression, whereas histone deacetylation is linked to transcriptional silencing. This dynamic regulation involves crucial enzymes, namely histone acetyltransferases (HATs) and histone deacetylases (HDACs). Studies have shown that various inhibitors of HDACs are effective in reactivating KSHV from latency. Sodium butyrate (NaB) and trichostatin A (TSA) are two well-known inhibitors of class I and class II HDACs. NaB promotes histone hyperacetylation, leading to significant chromatin remodeling at the KSHV RTA promoter, which enhances RTA expression and promotes KSHV reactivation. Furthermore, NaB also facilitates the rapid dissociation of LANA from the RTA promoter, thereby disrupting the interactions between LANA and cellular proteins Sp1 and histone H2B [55,56]. SIRT1 is a member of class III HDAC sirtuins (SIRTs) that preferentially deacetylates histone H3 at lysines 9 and 14 (H3K9 and H3K14) and histone H4 at lysine 16 (H4K16), leading to chromatin condensation and transcriptional repression [57]. During KSHV infection, SIRT1 can bind to the RTA promoter and inhibit RTA transactivation of its own promoter, preventing the expression of its downstream genes [58].

### 2.3. Post-Translational Modifications in KSHV Infection

#### 2.3.1. SUMOylation and KSHV Infection

Small ubiquitin-like modifier (SUMO) modification is one of the most well-characterized post-translational modifications, playing a crucial role in regulating various biological processes. In 2003, it was demonstrated that SUMO modification is associated with transcriptional repression, as core histone H4 undergoes SUMOylation both in vivo and in vitro. This SUMO-induced transcriptional repression depends on the recruitment of histone deacetylases and heterochromatin protein 1 (HP1) [59]. During KSHV infection, the viral protein KSHV basic leucine zipper (K-bZIP) promotes the SUMOylation of the histone demethylase Jumonji C (JmjC) domain-containing histone demethylase 2A (JMJD2A) in a SUMO-interacting motif (SIM)-dependent manner. This modification is crucial for JMJD2A’s binding to viral chromatin and plays a significant role in the transactivation of viral genes during KSHV reactivation [60]. In addition, SUMO is crucial for regulating the assembly and disassembly of PML-NBs or nuclear domain-10 (ND-10). PML-NBs serve as a vital component of the intrinsic antiviral defense by suppressing viral transcription and replication [61]. RTA is a SUMO-targeting ubiquitin-ligase (STUbL), capable of binding to SUMOs and mediating the degradation of PML, which in turn impairs the formation of PML-NBs [62]. Besides K-bZIP, LANA possesses two SUMO-interacting motifs (SIMs) that specifically bind to SUMO-2. This interaction enhances the SUMOylation of LANA. The SIMs in LANA recruit SUMO-modified chromatin remodeling proteins, such as Krüppel -associated box domain-associated protein-1 (KAP-1), leading to the silence of viral gene expression [20,63].

#### 2.3.2. Phosphorylation and KSHV Infection

Phosphorylation, a crucial post-translational modification, is activated by kinases or regulatory proteins and plays a fundamental role in regulating numerous physiological functions in eukaryotic cells. Viral protein kinase (vPK), encoded by KSHV ORF36, is a conserved serine protein kinase that induces both autophosphorylation and the phosphorylation of viral and cellular proteins. vPK can interact with and phosphorylate KSHV K-bZIP. This phosphorylation leads to a reduction in the SUMOylation levels and trans-repression activity of K-bZIP [64]. Moreover, vPK also induces the phosphorylation of KAP-1 and reduces KAP-1 SUMOylation, which in turn decreases the binding ability of KAP-1 on chromatin [65]. H_2_AX, an isoform of histone H_2_A, is significantly upregulated during KSHV infection. The phosphorylated form of H_2_AX interacts with LANA and plays a critical role in maintaining viral latency [66].

### 2.4. Chromatin Remodeling and KSHV Infection

Not only do histone modifications regulate gene transcription and expression, but chromatin remodelers do as well. Eukaryotic cells contain four families of chromatin remodelers, including swith/sucrose non-fermentable (SWI/SNF), imitation swith (ISWI), chromodomain helicase DNA-binding (CHD), and INOsitol requiring 80 (INO80). RTA, the transcription activator of KSHV, acts as a molecular switch for lytic reactivation that interacts with BRG1, a subunit of the SWI/SNF complex, and recruits the SWI/SNF complex to the promoter region to promote KSHV lytic reactivation [67,68]. In KSHV infected cells, NaB initiates KSHV reactivation. Mechanistically, NaB causes a significant increase in the association of Ini1/Snf5 with RTA and deregulates promoter-associated nucleosome [56]. In addition to RTA, K8 interacts and colocalizes with another chromatin-remodeling factor hSNF5 and promotes its transactivation activity [69].

### 2.5. Genome Conformation and KSHV Infection

KSHV genomic DNA is large and must exist within cells in a highly organized, folded state. This folding does not happen randomly but is well controlled by the structural maintenance of chromosomes (SMC) protein complexes. SMC protein complexes comprise cohesin, condensin and the SMC5/6 complex [70]. Cohesins mediate sister chromatin cohesion and CTCF (CCCTC-binding factor), and function at chromatin boundaries, playing key roles in the structural and functional organization of chromosomes [71]. During KSHV infection, CTCF and the cohesion complex associate at several regions on the KSHV genome, especially upstream of the major latency-associated transcript region [72]. CTCF and cohesion primarily facilitate the formation of two major chromatin loops in the KSHV episome. The first is a short 10 kb loop connecting a cluster of three CTCF sites upstream of the LANA ORF to the 3′ end of the K12 gene. The second is a larger loop (>50 kb), linking the CTCF binding site cluster to the promoter for RTA [73,74]. It has been suggested that the disruption of CTCF–cohesin binding eliminates the chromatin loops and reduces the expression of IE genes and increases LANA expression [74]. In addition, the knockdown of cohesion subunits Rad21, SMC1, and SMC3 promotes KSHV lytic gene transcription and viral DNA replication [75,76]. It is important to note that CTCF binding is sensitive to methylation at its binding site [77]. Therefore, DNA methylation on the KSHV genome is also expected to regulate CTCF binding and episome chromatin loops. These findings suggest that CTCF and cohesin are important for regulating latent and lytic viral gene expression. In addition to cohesin and CTCF, the SMC5/6 complex also plays an important role in regulating KSHV gene expression. The SMC5/6 complex can bind to the KSHV episome and suppress KSHV gene transcription by condensing the viral chromatin and creating a repressive chromatin structure [78].

### 2.6. Non-Coding RNAs

Non-coding RNAs (ncRNAs) encompass a group of RNAs that do not encode functional proteins and can be categorized into two main types: housekeeping ncRNAs and regulatory ncRNAs. Regulatory ncRNAs play a crucial role in epigenetic regulation and can be further grouped into two subclasses based on transcript size: small non-coding RNAs (20–200 nucleotides) and long non-coding RNAs (lncRNAs, over 200 nucleotides) [79]. Polyadenylated nuclear RNA (PAN RNA) is a viral lncRNA encoded by KSHV that significantly influences KSHV gene expression, replication, and immune modulation [80]. During the KSHV life cycle, PAN RNA is vital for the production of progeny virions and the expression of various lytic genes. Notably, a recombinant BACmid that deletes most of the PAN RNA locus fails to produce infectious viral particles and leads to the downregulation of the expression of RTA and other viral genes. Mechanistically, PAN RNA interacts with cellular H3K27me3 demethylases JMJD3 and UTX, erasing repressive marks on the KSHV genome and regulating viral gene expression [81]. In addition, PAN RNA enhances the interaction between ORF59 and JMJD3/UTX, further facilitating histone demethylation and promoting gene expression [54]. N4-acetylcytidine (ac4C) is a novel mRNA modification catalyzed by N-acetyltransferase 10 (NAT10) [82]. It has been suggested that NAT10 catalyzes ac4C addition to PAN RNA and triggers viral lytic reactivation from latency [83]. Mechanistically, PAN coordinates NAT10 and ATAT1 to enhance NAT10 lactylation, promoting the ac4C modification of tRNA Ser-CGA-1-1, and eventually it facilitates KSHV reactivation [84]. Another way PAN RNA may promote viral lytic reactivation is via the sequestration of LANA away from the KSHV episomes during reactivation [85].

MicroRNAs are a group of non-coding, small, single-stranded RNAs that inhibit gene expression by binding to the complementary regions of target genes, typically within the 3′ untranslated region (3′UTR). KSHV encodes 12 pre-microRNAs (pre-miRNAs) that are processed into 25 mature microRNAs (miRNAs) [86,87]. These microRNAs are located at the latent locus in the KSHV genome and are expressed during the viral latent phase [87]. Notably, some viral miRNAs can also present in viral particles and be transported into host cells [88]. These microRNAs are essential for regulating various important processes throughout the KSHV life cycle by directly targeting viral genes or indirectly influencing host cellular genes. Key processes modulated by these microRNAs include the maintenance of viral latency, the evasion of immune responses, and the regulation of cell proliferation and apoptosis [29]. For instance, miR-K9, miR-K7, and miR-K12-7-5p have been identified as direct regulators of RTA, suppressing its expression and helping KSHV in sustaining latent infection [36,89,90]. In addition to their direct effects on viral genes, various microRNAs also impact KSHV gene expression through modulation of specific cellular factors. MiR-K11, for example, regulates the transcription factor MYB, while miR-K3 influences the activity of nuclear factor I/B, both of which indirectly contribute to the suppression of RTA expression [91,92]. Furthermore, microRNAs can affect the methylation status of the RTA promoter. MiR-K4-5p inhibits the expression of retinoblastoma-like protein 2 (Rbl2), resulting in the upregulation of DNA methyltransferases 1, 3a, and 3b, promoting the methylation of the RTA promoter and facilitating the maintenance of latency in host cells [36]. SMAD5, an important mediator in TGF-β signaling, is downregulated by miR-K12-11 during KSHV de novo infection. The direct association between miR-K12-11 and SMAD5 inhibits cell proliferation [93]. Furthermore, it has been suggested that miRNAs such as miR-K5, miR-K9, and miR-K10 can trigger KSHV reactivation by repressing Bcl-2-associated transcription factor 1, which in turn inhibits apoptosis [94]. In addition to apoptosis, interferon (IFN) signaling is also important for medicating antiviral innate immunity. The ectopic expression of miR-K12-11 decreases I-kappa-B kinase epsilon (IKKε) expression, further inhibiting KSHV reactivation [95]. In summary, these KSHV-encoded microRNAs are crucial regulators of both KSHV latency and lytic replication, significantly influencing the virus’s ability to persist and propagate within host cells.

## 3. Transcriptional Regulation in KSHV Infection

**Figure 2:**
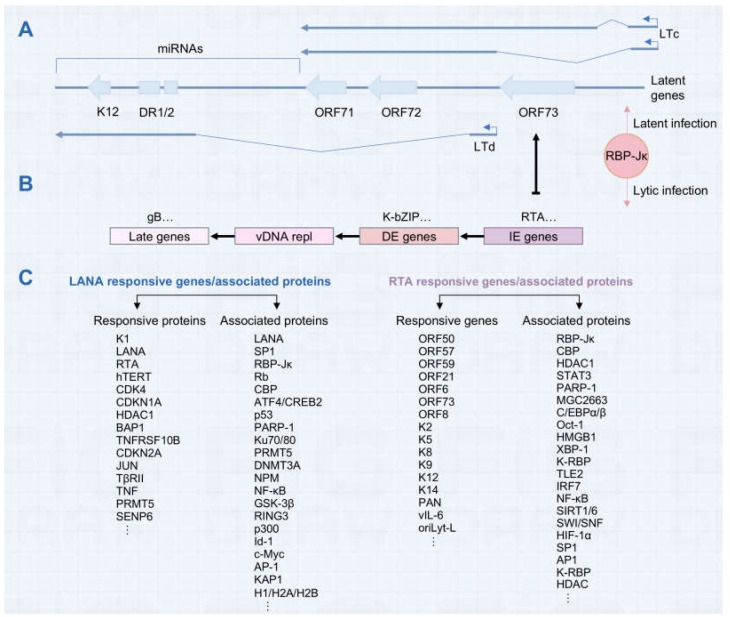
The schematic representation of gene expression and regulatory factors during the latency and lytic phases of KSHV infection. (**A**) A schematic depiction of the primary latency locus in KSHV. Middle panel: the four primary open reading frames (ORFs)-ORF73, ORF72, ORF71, and K12 alongside 12 pre-miRNA sequences are represented by vertical arrows in a light blue color. Top and Bottom panel: the transcriptional profiles directed by the Kaposin promoter (LTd) and the LANA promoter (LTc), respectively. (**B**) A schematic depiction of the sequential gene expression profile during the KSHV lytic phase is presented. The first set of genes expressed are referred to as the immediate-early (IE) genes, which includes RTA, etc. The second temporal class of mRNAs includes delayed-early (DE) genes, which includes K-bZIP, etc. Following the initiation of replication, late (L) genes are synthesized. These genes encode viral structural proteins, including gB, etc. RBP-jκ binding sites within the RTA promoter have been found to be critical for LANA-mediated repression. vDNA repl, viral DNA replication. (**C**) The identified LANA and RTA-responsive genes and associated proteins are shown.

Transcriptional control is an essential part of KSHV infection, governing the viral life cycle and impacting host interactions. Differences in gene expression between the latent and lytic phases of KSHV are critical to its capacity to maintain latency while sustaining lytic replication and pathogenicity. During latency, KSHV maintains its genome as an episome and only expresses limited viral transcripts encoded within the latency-associated gene cluster that facilitate persistence and immune evasion [9]. Among them, LANA as a functional viral protein is essential for the transcription of latent genes by binding to both viral and host promoters [96]. Additionally, host proteins such as Oct-1 [18] and Sp1 [97] can further facilitate the transcription of latency-associated genes, contributing to viral persistence. Upon reactivation, KSHV initiates a lytic phase defined by the expression of numerous lytic genes critical for viral replication and generation. The temporal gene expression program of KSHV during the lytic phase is a tightly regulated process that involves the sequential activation of immediate-early genes, early genes, and late genes [98,99]. The transcriptional regulation of KSHV entails a sophisticated and dynamic interplay between viral and host factors, underscoring the complexity of this process. The transcriptional regulation of KSHV is a multifaceted and dynamic process that relies on both viral and host factors. The delicate interplay is summarized in Figure 2.

### 3.1. Function of KSHV-Encoded Transcriptional Regulators

KSHV, a sophisticated virus, expresses around 90 proteins in an orchestrated temporal sequence across its replication cycle. This virus encodes an array of viral transcription factors crucial for governing its life cycle, particularly in modulating gene expression during both latency and lytic replication [100].

#### 3.1.1. LANA

LANA serves as a critical transcriptional regulator for KSHV by modulating the transcription of viral genes in several ways. Firstly, LANA can mediate transcriptional suppression and activation by direct DNA binding. In the presence of LANA, LANA binding sites 1 and 2 (LBS1 and LBS2) can repress transcription from an artificial minimal promoter when positioned upstream [101,102]. LANA binds to the promoters of latency-associated genes, facilitating their transcription while repressing RTA transcription and concurrently suppressing RTA expression, thereby contributing to the maintenance of KSHV in a latent state. It can directly repress transcription from the ORF50 promoter or indirectly repress it by competitively binding with co-factors CBP and the Notch pathway effector recombination signal-binding protein (RBP-Jκ), thereby preventing RTA-mediated auto-activation [24,25,103]. LANA can repress transcription of the K1 promoter by interacting with LBS1 and LBS2 in the transcriptional regulatory areas of the viral genome [104]. Beyond its specific DNA-binding capability, LANA functions as a versatile transcriptional co-factor on various promoters, regardless of the presence of its unique DNA-binding recognition motif. LANA can transactivate the human telomerase reverse transcriptase (hTERT) promoter by interacting with the cellular transcription factor Sp1. In addition, this is also achieved through interactions with a range of cellular proteins, including: RBP-Jκ [24], p53 [105], Rb [106], GSK-3β [107], CBP [108,109], ATF4/CREB2, Ring3, KSHV RTA [103].

In the context of KSHV infection, LANA plays a pivotal role in the transcriptional regulation of its own promoter. Even in the absence of other viral proteins, the LANA promoter (LANAp) remains active. However, when LANA is expressed, it further enhances the activity of its own promoter, establishing a positive-feedback mechanism [26,110,111]. This auto-activation not only sustains LANA’s expression but also contributes to the control of viral genome copy number and the expression of other viral genes [111]. Beyond its impact on the viral genome, LANA interacts with a variety of host transcription factors and chromatin-modifying proteins, thereby influencing the expression of host genes. It upregulates the expression of numerous cellular genes, including CDKN1A, CDK4, HDAC1, MCL1, BAP1, TNFRSF10B, and CDKN2A, JUN, and so on [112]. Recently, our group found that LANA upregulates PRMT5 in KSHV latently infected cells through transcriptional activation [113]. These interactions suggest that LANA has a broad role in modulating the host cellular environment to support viral persistence. Conversely, LANA also exerts inhibitory effects on certain host genes, such as the TGFβ-II receptor (TβRII) and TNF, modestly [33]. Our previous study also shown that LANA represses the expression of SUMO-specific peptidase 6 (SENP6), an enzyme responsible for protein desumoylation, thus promoting its own sumoylation and stability. This repression is crucial for maintaining KSHV latency, as sumoylation of LANA enhances its regulatory roles in episome maintenance and suppression of viral reactivation [114]. This dual capacity to both activate and repress gene expression allows LANA to fine-tune the host-cellular landscape, potentially promoting viral latency.

#### 3.1.2. vFLIP

Other KSHV latency genes, in concert with LANA, collectively contribute to the maintenance of KSHV in a latent state [115]. vFLIP and LANA collaborate to enhance the expression of enhancer of EZH2, the H3K27 methyltransferase component of the PRC2, by activating the NF-κB pathway [116]. Also, vFLIP attenuates RTA-mediated transactivation by modulating the NF-κB pathway, which in turn affects the binding and recruitment of RBP-Jκ [10,16]. This mechanism competes with RTA for the same co-factor, thereby reducing RTA’s ability to transactivate its target genes [14]. Furthermore, vFLIP is known to inhibit the AP-1 pathway, which is essential for KSHV lytic replication. By suppressing the AP-1 pathway, vFLIP can repress the transcription of RTA and other downstream lytic genes, thereby maintaining the viral latency [16]. This dual regulation of cellular pathways by vFLIP provides a means for KSHV to control its replication state and contribute to its ability to persist in a latent state within the host cell [117].

#### 3.1.3. RTA

KSHV ORF50 encodes a critical protein known as the replication and transcription activator (RTA). This protein is unique in its ability to induce the shift from viral latency to the active lytic phase singlehandedly [27,118,119]. Functioning as a transcriptional activator, RTA carries a DNA-binding domain that targets specific sequences on the DNA, along with a transactivation domain that boosts the transcription of genetic information [120]. The activation of RTA sets off a chain reaction that leads to the expression of other lytic genes, thereby orchestrating the viral replication process. This makes RTA not only a key player but also a central point of control in the transcriptional regulation of KSHV. Accumulation of RTA to a threshold level enables it to displace LANA from RBP-Jκ, its principal co-factor, thereby enabling RTA to bind to 100 specific sites within the viral genome [14,121]. This binding capability enables RTA to activate the transcription of 34 lytic genes, which are essential for the viral replication cycle. Among the genes transactivated by RTA are K8, K5, K2, K12, K14, ORF6, ORF57, ORF74, K9, ORF59, K3, ORF37, K1, K8.1A, ORF21, vIL-6, PAN RNA, vIRF1, and ORF65, as well as the two lytic replication origins, OriLyt-L and OriLyt-R, and a cluster of microRNAs [27,86,122,123,124]. RTA can achieve this regulation either directly, through RTA-responsive elements, or indirectly, by interacting with other viral or host regulatory factors, thereby orchestrating a comprehensive transcriptional program that drives the lytic phase of the viral life cycle [122,125,126,127].

Transcriptional regulation by RTA on downstream gene promoters is intricate and complex. Broadly speaking, the precision of RTA in targeting specific promoters is attributed to its dual mechanism of action, which can be either direct or indirect. Furthermore, the RTA’s engagement with these promoters is flexible, operating independently or in conjunction with the cellular factor such as RBP-Jκ [126]. Direct transcriptional activation is achieved through RTA binding to RTA-responsive elements (RREs) in certain promoters. RREs are specific DNA sequences that serve as binding sites for RTA, allowing it to directly activate the transcription of downstream lytic genes, effectively driving the virus from latency into a productive lytic state [128,129]. Several replication and transcription activator response elements (RREs) have been identified in the promoters of delayed-early viral genes (e.g., tk, ORF57/MTA, K8/RAP, K2, K9, K14, and DBP) and in one late promoter (gB) using a promoter–reporter cotransfection assay. RTA also activates another set of target genes, PAN and K12, via a direct DNA-binding method. These genes feature RTA response elements in their promoter regions that are similar in sequence to those found in the viral lytic replication origin, oriLyt-L, which is situated between the ORFs K4.2 and K5 [128]. Notably, RTA’s interaction with the vIL-6 promoter, despite its ability to bind, shows little sequence resemblance to the conserved sequences present in the PAN and K12 promoters, suggesting a divergence in the mechanisms of RTA-mediated gene activation across different viral genes [130]. The reason for the RTA’s diverse DNA-binding specificities is unclear. Additionally, ORF50 induces the expression of numerous target genes via an indirect way. Among the various co-factors that work with RTA, the protein RBP-Jκ (also known as CSL), a crucial component of the Notch signaling pathway, is particularly essential. It plays an indispensable role in enabling RTA to activate transcription at both viral and cellular promoters and is vital for efficient viral reactivation. Typically, RBP-Jκ binds to DNA in promoter regions and functions as a repressor until activated by signaling cues. The interaction with RTA converts RBP-Jκ from a repressive to an activating state for target gene transcription. Such transactivation by the ORF50 protein has been documented for several promoters, including those of ORF57, ORF6, K14/ORF74, K6 (vMIP-1), ORF50, and LANA (LTi) [24,131,132,133,134]. Furthermore, the RTA’s action through protein–protein interactions with RBP-Jκ suggests an indirect mechanism that may proceed independently of RTA’s DNA-binding function yet is contingent upon the expression of RTA [134]. Besides RBP-Jκ, other cellular proteins, such as C/EBPβ [135], OCT1 [18], c-Jun [136], Sp1 [15], etc., have been shown to interact with ORF50-responsive promoters.

Moreover, RTA can auto-stimulate its own promoter, potentially amplifying subtle environmental signals to facilitate viral replication [124]. Within the promoter region of RTA in KSHV, three principal sets of regulatory elements—the Oct1, C/EBP, and RBP-Jκ binding sites—are implicated in the autoregulatory control of RTA [18,132,135]. Furthermore, six RBP-J binding sites are identified in the ORF50 promoter [131,132]. The interaction of RBP-Jκ with these sites is presumed to be essential for the autoregulatory control of RTA, suggesting a key mechanism by which the gene modulates its own transcriptional activity [137]. RTA initiates a positive feedback loop by transactivating its own promoter, which surpasses latency and amplifies lytic gene expression. This loop is perpetuated by lytic gene products: the K8 protein stabilizes CEBPα to enhance RTA-mediated transactivation, while ORF57 binds to RTA, boosting transcription from the RTA promoter [119,135,138]. Consequently, the interplay between lytic proteins sustains KSHV reactivation and propels the lytic cycle forward [115]. RTA also positively regulates all KSHV lytic enhancers [139]. RTA activates the terminal repeats (TR) enhancer, while LANA represses this enhancer to maintain the latency cycle.

#### 3.1.4. K-bZIP

The KSHV-encoded protein K-bZIP, derived from the K8 gene, exerts substantial influence over the regulation of viral gene expression. This protein also plays a crucial role in the virus’s capacity to adjust the host cell’s responses, thereby contributing to the intricate interplay between the virus and its host. In a screen of 83 potential KSHV promoter regions within 293 cells, the K-bZIP protein was found to independently activate 21 promoters. Additionally, it enhances the activation of 19 promoters in conjunction with RTA, while it also suppresses the RTA-mediated transactivation of 3 promoters [140]. Direct protein–protein interaction is attributed to the inhibition of RTA transactivation by K-bZIP; hence, the overexpression of transcriptional coactivators is not helpful to offset this inhibitory effect [28].

### 3.2. Function of Host Transcriptional Regulators

The host transcription factors that regulate KSHV gene expression include a diverse set of proteins. A number of cellular DNA-binding proteins, along with transcriptional co-factors and enzymes, have been demonstrated as essential for controlling the expression of both latent and lytic genes in KSHV. These proteins play a pivotal role in the virus’s ability to switch between its dormant and active states, thereby influencing the viral life cycle and pathogenesis, including response element-binding protein (CREB) [141], p53 [142], CREB-binding protein and p300 (CBP/p300) [141], RBP-Jκ [134], CCAAT/enhancer-binding protein (C/EBP) [119], interferon regulatory factor 7 (IRF7) [143], specificity protein 1 (SP1) [15], octamer-binding transcription factor 1 (OCT1) [18], activator protein 1 complex (AP-1) [16], NF-κB [14], hypoxia-inducible factor 1-alpha (HIF-1α) [144], c-Myc [145], signal transducer and activator of transcription 3 (STAT3) [17], GATA [146], E2F [106], RUNX3 [23], cAMP [109], histone deacetylase-1 (HDAC1) [141], MGC2663 [147], SWI/SNF complex, TRAP/mediator complex, poly(ADP-ribose) polymerase 1 (PARP-1) [148], Ste20-like kinase (hKFC). As above mentioned, numerous host factors participate in the transcriptional modulation of KSHV. Below, we will emphasize some key host transcriptional regulators that operate during KSHV’s latent and lytic stages.

LANA is essential for initiating KSHV latency, with its multifaceted roles relying on its capacity to recruit host factors to the viral genome. For example, LANA binds to both N- and C-terminal domains of KAP1, recruiting it to the RTA promoter and terminal repeat (TR) regions of the KSHV genome. This interaction leads to transcriptional repression of lytic genes, helping to establish and maintain viral latency [20]. Moreover, LANA induces protease-mediated cleavage of STAT6, removing its transactivation domain and converting it into a transcriptional repressor of the RTA promoter [149]. Another study proved that LANA interacts with and suppresses the activity of both Rb [106] and p53 [150] in experimental assays. Inversely, p53 has been shown to suppress the activity of the LANA promoter, indicating a reciprocal regulatory relationship between LANA and these cellular proteins [111]. Notably, NF-κB also plays a key role in maintaining KSHV latency by preventing the expression of lytic genes. The viral protein vFLIP continuously activates NF-κB signaling to support this latent state [151]. KSHV lytic action is competitively inhibited by activated NF-κB complexes with the RTA co-factor RBP-Jκ [14]. However, NF-κB’s function is complex, as it can also promote viral reactivation under specific conditions. This context-dependent regulation highlights the multifaceted role of NF-κB in KSHV infection. In the context of KSHV infection, STAT3 is also constitutively activated to modulate KSHV latency and lytic replication through influencing the expression of key viral genes. For example, STAT3 has been shown to interact with the KSHV genome, particularly with the regulation of the viral interleukin-6 (vIL-6) gene, which is crucial for viral replication and immune evasion. Furthermore, STAT3 has been found to repress the expression of RTA, and therefore it helps to maintain the virus in a latent state, preventing premature lytic replication and the associated cellular damage that could trigger an immune response [152,153]. Additionally, KSHV uses Sp1 binding sites on the promoters of latency genes, such as LANA, to maintain latency. Likewise, Sp1 contributes to the regulation of viral lytic genes during reactivation as well, thereby serving as a bridge between the latent and lytic phases. Our group’s research has demonstrated that LANA interacts with KAP1 and directs it to the RTA promoter within the KSHV genome. This interaction plays a role in the suppression of lytic gene expression by LANA [20]. A number of studies have indicated that LANA recruits multiple cellular transcription factors, including SP1 [97], E2F, Ap1, RBP-Jκ, ATF4 [108], CBP, Id-1 [154], and nucleosome assembly protein (NAP1L1) [155], and influences their regulation of viral gene expression. Additionally, the enzymes poly (ADP-ribose) polymerase 1 and Ste20-like kinase hKFC function as transcriptional repressors, controlling the lytic replication of gamma-2 herpesvirus [148]. Furthermore, the activated form of Nrf2 collaborates with LANA and KAP1 to suppress the expression of lytic genes, indicating its role in maintaining KSHV latency [156].

During KSHV latency, RBP-Jκ is associated with the repression of lytic gene expression. Upon activation signals, RBP-Jκ can dissociate from the repressive complexes, allowing for the transcription of lytic genes [157,158]. This switch is critical for the transition from latency to lytic replication. RBP-Jκ can also recruit co-activators such as CBP/p300 to KSHV promoters, which enhances transcriptional activity [159]. Additionally, NF-κB acts as a key cellular regulator during KSHV infection, sensing viral latency and inhibiting the switch to the lytic phase. It achieves this by counteracting the activity of RTA. Specifically, NF-κB interferes with RTA’s ability by antagonizing the function of RBP-Jκ, a coactivator required for RTA’s transcriptional activity [14]. In addition to RBP-Jκ, RTA also engages with C/EBPβ and STAT3 to stimulate the activity of both viral and cellular promoter [17]. Additionally, SIRT1 has been shown to physically associate with RTA, thereby inhibiting the activation of its own promoter as well as other lytic gene promoters. Similarly, SIRT6 binds to the KSHV ori-Lyt and ORF50 promoters, repressing viral replication by interacting directly with the KSHV genome and blocking viral gene expression [115]. Several other proteins are involved in forming an active transcriptional complex with RTA’s C-terminal transactivation domain. These include the CBP histone acetyltransferase, the BRG1 component of the SWI/SNF chromatin remodeling complex, and the TRAP230 subunit of the TRAP/mediator complex, which may be crucial for RTA-mediated transcriptional activation [67,141]. Protein kinase C (PKC) delta isoform activates the MAPK/ERK pathway upon treatment with inducers such as TPA, which in turn leads to the phosphorylation of c-Jun and accumulation of c-Fos. This activation forms an active AP-1 complex, which is crucial for initiating the transcription of the KSHV RTA gene and subsequently triggering the lytic replication cascade [160]. Another signaling pathway, Raf/MEK/ERK/Ets-1, is instrumental in Ras-induced RTA activation. The activation of Ras leads to the transcription of downstream genes like PAN, kaposin, ORF57, and vIL-6, which contribute to the lytic cycle of KSHV [161]. In hypoxic conditions, HIF-1α and HIF-2α activate the RTA promoter directly through hypoxia response elements. This promotes KSHV reactivation under low oxygen conditions, which is commonly observed in Kaposi’s sarcoma lesions [144]. Recently, our group found that RUNX3 is upregulated at the transcriptional level by the NF-κB signaling pathway in KSHV-infected SLK cells and B lymphocytes during the reactivation of KSHV. Functionally, RUNX3 interacts with the KSHV genome and impairs viral replication via a mechanism of transcriptional repression, which is linked to its capacity to bind DNA and ATP [23].

## 4. Future Perspectives

There is substantial evidence indicating that the establishment of latency and the reactivation of KSHV are intricately connected to both epigenetic and transcriptional regulation. However, the mechanisms by which these regulatory processes influence the KSHV genome are intricate and dynamic. The complex interplay of both positive and negative epigenetic regulation of KSHV transcription is inextricably linked to the various stages of the KSHV life cycle. During latency, the viral genome is maintained in an epigenetically repressed state, characterized by limited gene expression, allowing for episomal replication while simultaneously preventing immune activation. Under some certain conditions, the fate of KSHV is rewriting. The epigenetic and transcriptional repression of the viral genome is alleviated, and the viral chromatin structure is maintained in an active state, resulting in an increased expression of various lytic viral proteins. This complex epigenetic and transcriptional program requires a variety of epigenetic modifications and a plethora of transcriptional regulators within the host cell. However, what drives, organizes, and regulates such an orderly, dynamic, and precise process remains to be further explored. It is important to visualize the association between the KSHV genome and cellular epigenetic factors. Specifically, demonstrating the dynamic binding of chromatin remodeling factors and SMC complexes to the KSHV genome is essential for understanding how KSHV maintains latency or reactivates into lytic replication. Rapidly advancing genome-wide and spatial sequencing approaches with increased resolutions will significantly enhance our understanding of the epigenetic modifications in the KSHV viral genome. In addition, a more focused investigation into the binding characteristics of transcription factors associated with KSHV is crucial. Currently, we lack a comprehensive characterization of certain key regulatory factor-binding sequences within the KSHV genome. For instance, the RTA response elements within the vIL-6 promoter do not share significant similarity with the conserved sequences found in the PAN and K12 promoters [130]. However, rapid advancements in bioinformatics and structural analysis techniques have greatly enhanced our ability to handle large-scale complex datasets. This progress enables us to systematically describe and summarize these binding characteristics, providing substantial support for our efforts to combat KSHV-related diseases.
